# The Effects of Humidity and Seasonality on Foliar and Root Mycobiomes of *Betula pendula*

**DOI:** 10.1111/1758-2229.70145

**Published:** 2025-07-07

**Authors:** Mihhail Brodski, Arvo Tullus, Ahto Agan, Kalev Adamson, Rein Drenkhan, Katrin Rosenvald, Arne Sellin

**Affiliations:** ^1^ Department of Botany, Institute of Ecology and Earth Sciences, Faculty of Science and Technology University of Tartu Tartu Estonia; ^2^ Chair of Forest Ecology and Management, Institute of Forestry and Engineering Estonian University of Life Sciences Tartu Estonia

**Keywords:** air humidity, boreal forest, climate change, endophytes, foliar fungi, silver birch

## Abstract

An increase in precipitation is predicted for Northern Europe over the coming century. In order to research how this change may affect fungi growing on Silver birch ( *Betula pendula* ), samples of its leaves and fine roots were collected over a 3‐year period in the Free Air Humidity Manipulation experimental area in Estonia. Fungal DNA was extracted from the samples and sequenced to analyse the fungal communities. We found that increased air humidity shifts fungal community composition, but elevated soil irrigation does not. Variability in fungal communities caused by the experimental treatment was smaller than variability between sampling years and months. The response of species richness to elevated air humidity was inconsistent, whilst the proportion of saprotrophs and fungal endophytes increased. Analyses revealed that around 16% of all fungal species present in birch leaves avoided elevated air humidity, less than 2% preferred it. Fine root fungal communities appeared to be somewhat less influenced by changes in atmospheric humidity. Our results suggest that most of the foliar fungi are rather resilient to changes in atmospheric humidity, with a minority of species being sensitive to it, but the birch foliar fungal community as a whole does undergo a shift in composition.

## Graphical abstract

We conducted a DNA metabarcoding study in which we investigated how elevated air humidity, an effect of climate change predicted for Northern Europe, may influence the communities and ecological functions of fungi growing in silver birch ( *Betula pendula* Roth) foliage and fine roots.

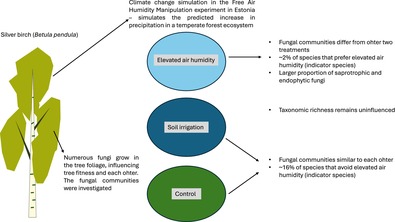

## 1 Introduction

Silver birch ( *Betula pendula* Roth) is a fast‐growing and shade‐intolerant tree species that is widespread in Eurasia and is one of the dominant species in northern temperate and boreal forests (Hynynen et al. 2010). In hemiboreal forests of Estonia, it makes up, together with the downy birch ( *Betula pubescens* Ehrh.), 26% of forest growing stock (Valgepea et al. 2022). Silver birch is of great economic importance, being the main source of hardwood in northern Europe (European Commission 2016) and of great ecological value, growing on a wide variety of forest types and on a wide range of soils as well as being used in forest restoration, e.g., on former agricultural soils (Hynynen et al. 2010; Lutter et al. 2023). Silver birch has become a model tree for studying climate change impacts on forests in northern Europe (Anger‐Kraavi et al. 2021; Oksanen 2021) due to its high plasticity and great potential acclimation ability.

Both foliage and roots of silver birch host communities of plant‐associated fungi that are still little explored. The foliage is a rather hostile and oligotrophic environment that is consistently exposed to fluctuating weather conditions and different stressors. It has been claimed that only about 8% of fungi manage to sporulate on birch leaves (Warren 1976). This, coupled with active defence mechanisms of the plant, makes the foliar fungal communities less species‐rich than fungal communities in the soil (Lebeis 2015) and temporally more variable (Sieber 2007). A large part of the fungi present in the phyllosphere is believed to be generalist species capable of living on many different plants. However, oftentimes the foliar fungal communities are dominated by a few host‐specific species (Sieber 2007), with numerous other species being present as well. Classifying fungal–plant relations is convoluted by plasticity in lifestyles of fungi, as their life history may change depending on host species or even its health and environmental conditions (Moricca and Ragazzi 2011) and due to that, the exact functions of the core plant mycobiome have not yet been properly described.

Plant root‐inhabiting endophytes as well as mycorrhizal fungi very often help plants to adapt to environmental stresses, such as drought (De Zelicourt et al. 2013) and excess salinity. Numerous studies have investigated questions related to the mitigation of environment‐caused plant stress by fungi, providing a good base to assume that plant‐associated fungi will be important benefactors for plants to adapt to climatic changes (Kivlin et al. 2013). Soil and plant root microbial communities are known to be influenced by soil properties such as pH, nutrient availability and water content as well as the plant species and their root traits (Lauber et al. 2008; Nakayama et al. 2019). In turn, these microbial communities drive nutrient cycles in soil (Isobe et al. 2018; Štursová et al. 2012). It has been shown earlier that birch trees grow longer and thinner absorptive roots in elevated air humidity conditions (Parts et al. 2013; Rosenvald et al. 2014). In addition, ectomycorrhizal fungal communities also appear to change due to the increase in air humidity (which prolongs periods with high soil moisture due to decreased evapotranspiration), with hydrophilic fungal morphotypes becoming more abundant (Parts et al. 2013), which reflects their adaptation to wetter soil conditions.

The phyllosphere microbiome is known to change over time—both cyclical seasonal changes and long‐term changes are present. It has been shown that seasonal changes in communities are bigger than those caused by short‐term weather fluctuations both in phyllosphere and plant roots (Nakayama and Tateno 2023; Stone and Jackson 2021). Seasonal changes in foliar microorganism communities can be drastic, but they often remain similar from year to year and microbial communities remain active even in dormant seasons (Isobe et al. 2018). In birches and many other deciduous trees, the leaves start almost inhabitant‐free in the beginning of the growing season, and gradually more and more fungi and bacteria start inhabiting the leaves, with peak colonisation density happening shortly before leaf senescence (Sieber 2007; Agan et al. 2020). So far, very few long‐term studies spanning several years have been conducted to investigate the long‐term changes in foliar fungal communities and, to our knowledge, none have so far been done in birch species. However, without a doubt, climatic conditions as well as sudden events can be long‐term drivers of foliar fungal communities (Zhu et al. 2022). Still, the complexity and dynamism of phyllosphere as an environment, and fungal and microbial communities inhabiting there will make it difficult to comprehensively describe and predict the effects of climate changes on composition and functioning of these communities. Because of that and a relatively recent emergence of this study field there are currently also no model systems describing the assembly of phyllosphere microbiomes.

The diversity and composition of the horizontally transmitted foliar fungi are influenced both by biotic factors and abiotic environmental conditions, such as humidity, air temperature, solar radiation and soil type. Predictions of near‐future climatic changes state that an increase in annual average precipitation and relative air humidity is expected in the Baltic Sea region (Anger‐Kraavi et al. 2021; Christensen et al. 2022; Kjellström et al. 2018). In this study, we sampled leaves and fine roots of silver birch trees that are growing in the Free Air Humidity Manipulation (FAHM) experimental area that was created in south‐eastern Estonia in order to research the effects of increasing precipitation and air humidity on forest ecosystems (Kupper et al. 2011). Our aim was to investigate how the experimental treatments of elevated relative air humidity and increased soil moisture would affect the foliar fungal community composition and species richness in silver birch leaves and fine roots and whether these effects would be consistent over the years and different seasons (summer versus fall). We hypothesised that elevated relative air humidity promotes the development of a more species‐rich foliar mycobiome, which may be more harmful to plant health, whilst in the roots, where there is usually no shortage of available water, the effect of increased humidity would be negligible.

## 2 Materials and Methods

### 2.1 Study Area and Sample Collection

**FIGURE 1:**
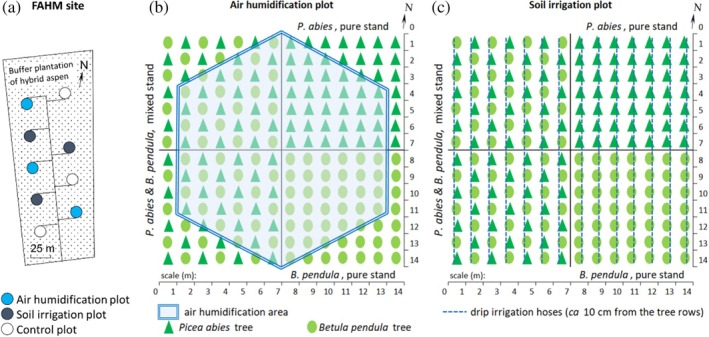
Outlines of the FAHM research area (a), air humidification (b) and soil irrigation (c) plots; control plots have a similar arrangement of trees.

**FIGURE 2:**
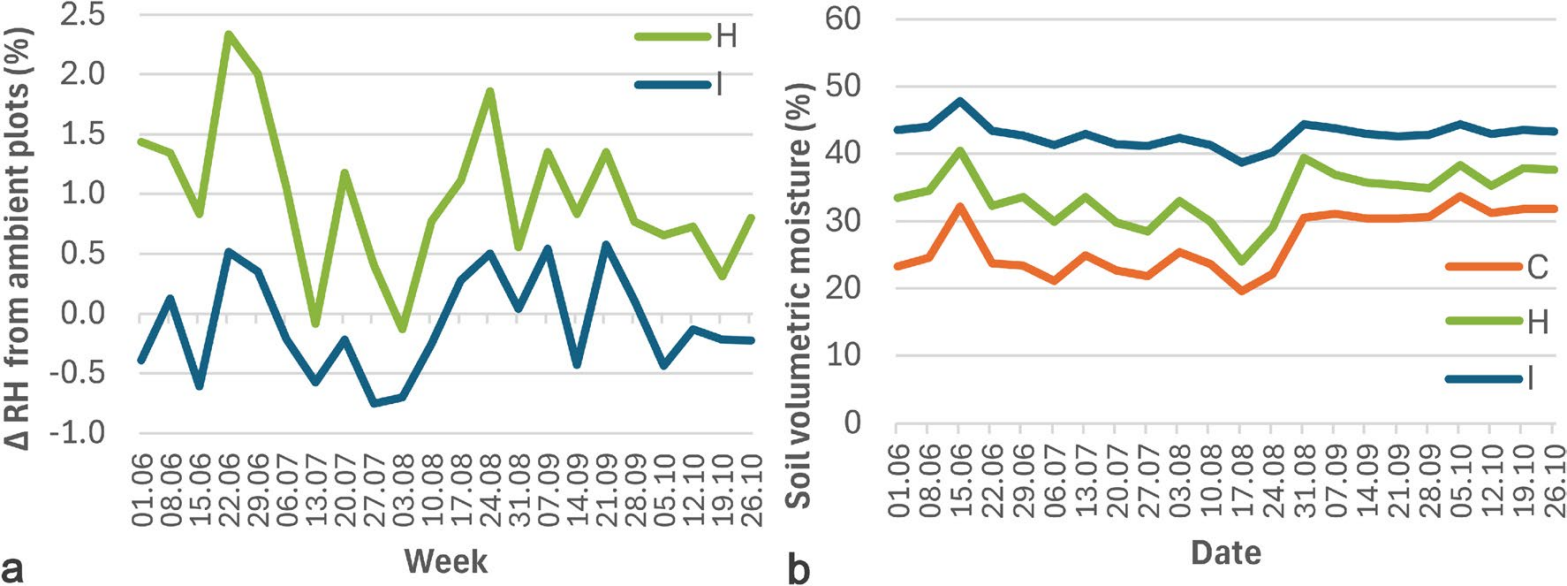
Difference in relative humidity (ΔRH) between the irrigation (I) and humidification (H) treatments and ambient conditions of control (C) treatment (a) and soil volumetric moisture content in different treatments (b) in 2022.

The study was performed at the Free Air Humidity Manipulation (FAHM) experimental site, which is located in Rõka village, Estonia (58.2455° N, 27.2995° E). The experimental site contains nine plots (Ø 14 m, Figure 1a), three of which are control plots (C), where air humidity is ambient; three are air humidification plots (H), where relative air humidity is elevated by 2%–3% (Figures 1b and 2a); and three are soil irrigation plots (I), where 15% of water is added to the previous week's actual precipitation (Figures 1c and 2b). Each plot is divided into four quarters, one quarter contains a pure silver birch stand, one contains a pure Norway spruce ( *Picea abies* ) stand, and two contain birch‐spruce mixed stands with the tree species in a 50:50 proportion. Two‐year‐old birches and 3‐year‐old spruces were planted in the spring of 2020 with 1 × 1 m spacing. Hybrid aspen (*Populus tremula L ×* *P. tremuloides* Michx.) trees grow in the buffer zones surrounding the experimental plots.

Based on the automated weather station of FAHM, the mean annual temperature and total annual precipitation in the study years were: 8.0°C and 582 mm (2020), 6.4°C and 556 mm (2021) and 6.8°C and 608 mm (2022). Compared to the long‐term (1991–2020) means of 6.0°C and 654 mm (average of the two nearest stations of the Estonian Environment Agency), all study years were somewhat warmer and drier, with 2021 having a dry period in June and July.

The leaf samples were collected twice a year (in July and September) from randomly selected trees from 2020 to 2022. The root samples were collected once a year (in October) in 2021 and 2022. We collected the leaf samples from the middle part of the tree crowns. From every plot we took six birch leaf samples, each time choosing two random trees from the pure birch stand section and two from each of the mixed stand sections. As a result, we had 18 samples per treatment in July and September, totalling 108 samples per year and 324 samples over 3 years of collecting. After collecting, the samples were immediately frozen at −20°C. The root samples were collected from one tree in each of the three quarters in every plot. Hence, nine root samples per treatment were collected, totalling 27 samples per year and 54 samples over 2 years. After collecting, the root samples were thoroughly washed of all the soil with water, dried at 40°C, and then frozen at −20°C.

### 2.2 DNA Extraction and Molecular Analysis

Half a birch leaf without the petiole was used for the extraction of DNA. From root samples, 0.2–0.25 g of dried fine roots were taken. The DNA from the collected samples was extracted using the GeneJET Genomic DNA Extraction Kit (Thermo Fischer Scientific Vilnius, Lithuania), following manufacturers' instructions, with some modifications made to the protocol as described by (Drenkhan et al. 2017). For amplification of fungal DNA both from leaves and roots, universal fungal primers were used, ITS1catta as the forward primer (Tedersoo and Anslan 2019) and ITS4NGSUni as the reverse (Tedersoo et al. 2014). All the samples were sequenced using the PacBio sequencing platform. We used the same primer pair for both leaf and root samples in order to reduce potential amplification biases, where some taxa can be over‐ or underrepresented due to using different primers within the same study.

Conventional PCR analysis was done twice per sample. PCR product was 25 μL in volume, containing 0.5 μL of both primers, 5 μL of HOT FIREPol Blend Master Mix (Solis BioDyne, Tartu, Estonia), 1 μL of DNA and 18 μL of water. The thermocycler for DNA amplification was set up as follows: 15 min at 95°C, followed by 25 cycles of 30 s at 95°C; 30 s at 55°C; 1 min at 72°C, and a final step of 72°C for 10 min. Positive and negative controls were used throughout the analysis to account for tag switches and possible sample contamination during the PCR process.

The PCR reactions were checked for the presence of PCR product on 1% agarose gel. In case of no visible band, amplification was repeated, with increasing the number of cycles to 35. The PCR products were purified using FavorPrep GEL/PCR Purification Kit (Favorgen, Vienna, Austria) following the manufacturer's instructions.

### 2.3 Bioinformatics

Bioinformatic analysis was performed using various programmes available via PipeCraft 2.0 (Anslan et al. 2017). The raw DNA sequencing data containing sequences longer than 100 bp was demultiplexed, allowing up to three nucleotides of mismatch during the index search. Then, cutadapt (Martin 2011) was used to remove primer sequences from data files. Quality filtering and de‐novo chimaera filtering were performed by vsearch v.2.23.0 (Rognes et al. 2016). Full and partial ITS regions were extracted from the sequence files with ITSx (Bengtsson‐Palme et al. 2013). Reads were clustered into operational taxonomic units (OTUs) using vsearch v2.23.0 (Rognes et al. 2016) based on a 98% similarity threshold, and post‐clustering was performed with luluv0.1.0 (Frøslev et al. 2017) with a minimum similarity threshold of 90%. Taxonomy to OTUs was assigned using BLAST 2.14.0+ (Camacho et al. 2008) against the UNITE v.9 database (Kõljalg et al. 2013). OTUs were considered members of Fungi if their representative sequences matched best fungal taxa at an *e*‐value of <e‐60. OTUs with a similarity coverage of less than 70% to the database references were removed from the dataset. OTUs with the same species assignment were manually summarised into one using LibreOffice Calc software.

### 2.4 Statistical Analysis

Testing the significance of dissimilarities between fungal communities in different treatments and the analysis of community variation partitioning were performed using, respectively, the anosim and adonis2 functions (using Bray‐Curtis dissimilarities) of the vegan package (Oksanen et al. 2001) in R (R Core Team 2023). Anosim was used for pairwise comparisons between communities responding to experimental and temporal factors, and adonis2 to evaluate the importance of various factors in shaping the fungal communities. To visualise the differences between communities, NMDS analyses were performed using the vegan package metaMDS function, and corresponding plots were created using the ggplot2 package (Wickham et al. 2007). The envfit function of the vegan package was used to test the significance and strength of correlation of environmental variables to the NMDS ordination (goodness of fit). Indicator species analysis was performed using the indicspecies package (De Cáceres et al. 2010) in order to test individual OTU's responses to the experimental treatments. Cumulative data over all sampling points was used for this test. Taxa indicative of two treatments at once indicated that these taxa were common in two distinct treatments but mostly absent in the third.

To investigate the impact of experimental treatments on species richness we employed linear mixed effects model (LMM) for repeated sampling, where plot and tree ID were included as random factors. The fixed factors were treatment (C, H, I), sampling month (July for mid‐season or September for late‐season) and sampling year (2020, 2021, 2022). The models had a full‐factorial design, i.e., included up to 3‐way interactions of fixed factors. The dependent variable was non‐transformed OTU richness in a sample. LMM analysis was performed with function lmer from the lme4 package (Bates et al. 2015). Proportions of various functional groups (pathogens, saprotrophs, endophytes and fungi with unknown ecology, mostly fungi assigned to higher taxonomic levels), as provided in the FungalTraits database (Põlme et al. 2020) were tested as probabilities using logistic regression with binomial distribution (using glmer function of lme4 package). *P*‐value of 0.05 was used as a cutoff for statistical significance. We defined endophytes as any fungi that are marked as having endophytic capacity in the FungalTraits database. The model residuals were inspected visually for their compliance with assumptions of normal distribution and homogeneity of variance.

## 3 Results

### 3.1 Bioinformatic DNA Sequence Classification

After bioinformatics processing, the resulting birch leaf OTU table contained 755 OTUs in total across 318 samples; the birch roots table contained 294 OTUs across 49 samples. The amount of OTUs in leaf samples varied from 3 to 160 (in two samples); in roots from 1 (in two samples) to 81. The 5 taxa comprising the most sequences in leaf samples were *Phyllactinia guttata* (24.1% of all sequences), an unidentified *Dothideomycetes* sp. (9.9%), a *Taphrina* sp. (8.5%), *Phialophora cerealis* (6.3%) and a *Ramularia sp*. (5.6%). In root samples, the most abundant sequences were those of *Tomentella sp*. (14.8% of all sequences), *Serendipita* sp. (10.7%), *Legaliana badia* (8.2%), *Phialocephala* sp. (5.6%) and *Peziza sp*. (4.9%).

In birch leaves, 89.2% of all sequences belonged to Ascomycota, 9% to Basidiomycota, and 1.8% were Fungi identified only at kingdom level. The rest of the sequences belonged to Rozellomycota, Mucoromycota, Mortierellomycota and Oomycota of Stramenopile kingdom. The most abundant fungal classes were Dothideomycetes (39.9% of all sequences), Leotiomycetes (25.5%), Taphrinomycetes (15.3%), Eurotiomycetes (6.6%), Tremellomycetes (5.3%), and unidentified Ascomycetes made up 1.4% of all sequences.

In root samples, 46.8% of all sequences belonged to Basidiomycota; 45.2% belonged to Ascomycota; 7.4% were fungi identified only at kingdom level. 0.5% belonged to Mortierellomycota; the rest belonged to Mucoromycota, Oomycota; Glomeromycota was present in one sample, possibly from soil contamination. The most abundant classes were Agaricomycetes (44.7% of all sequences); Pezizomycetes (19.3%); Leotiomycetes (16.3%); Sordariomycetes (5.9%); Dothideomycetes (2.4%) and Tremellomycetes (1.9%).

### 3.2 Explanatory Power of Environmental Factors

The variation in fungal communities in birch leaf and root samples was determined using PERMANOVA analysis. In leaf samples, the variation was explained by month of sample collection (*R* ^2^ = 0.108, *p* < 0.001), year of sample collection (*R* ^2^ = 0.098, *p* < 0.001), the experimental treatment (*R* ^2^ = 0.065, *p* < 0.001), and sampling plot (*R* ^2^ = 0.017, *p* = 0.010). In root samples, the variation was explained by the experimental treatment (*R* ^2^ = 0.053, *p* < 0.001) and year of sample collection (*R* ^2^ = 0.045, *p* < 0.001).

**FIGURE 3:**
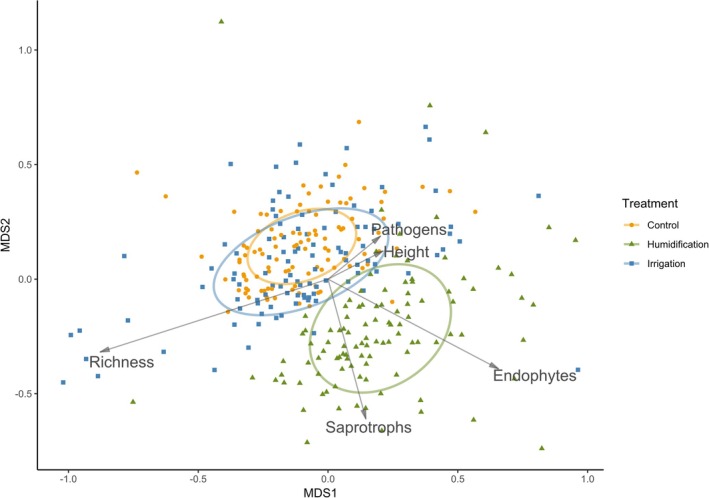
NMDS ordination biplot of fungal communities in silver birch leaves, stress = 0.259. Goodness of fit of various factors to the ordination: Experimental treatment *r* ^2^ = 0.253, *p* = 0.001; species richness *r* ^2^ = 0.444, adjusted *p* = 0.004; pathogen percentage *r* ^2^ = 0.039, adjusted *p* = 0.01; endophyte percentage *r* ^2^ = 0.288, adjusted *p* = 0.004; tree height at the end of the year of sampling *r* ^2^ = 0.03, adjusted *p* = 0.030. The ellipses represent the 95% confidence area of fungal communities in the treatments.

Using ANOSIM analysis we found out that there were significant differences in birch leaf mycobiome communities between the treatments (ANOSIM *R* = 0.163, *p* = 0.001); the communities in H treatment differed from both C and I treatments, but there were no differences between C and I. Significant differences in foliar fungal communities were between different sampling years (ANOSIM *R* = 0.225, *p* = 0.001); pairwise comparisons revealed differences between all sampling years (Table 1). Difference in leaf fungal communities between sampling months was also detected (ANOSIM *R* = 0.354, *p* = 0.001, Table 1). Based on NMDS ordination, taxonomic richness was strongly correlated with community composition (Figure 3). Taxonomic richness was negatively correlated with sampled tree heights and the proportion of pathogenic species in samples. Fungi with endophytic capabilities and saprotrophs made up larger proportions of fungal species richness in H treatments.

**TABLE 1:** Pairwise ANOSIM comparisons of fungal communities in both leaf and root samples.

Leaves			Roots		
Comparison	ANOSIM R	Adjusted *p*	Comparison	ANOSIM R	Adjusted *p*
C vs. I	0.005	0.160	C vs. I	0.08	0.083
C vs. H	0.276	**0.003**	C vs. H	0.15	**0.011**
H vs. I	0.201	**0.003**	H vs. I	−0.012	0.6
2020 vs. 2021	0.3	**0.001**	2021 vs. 2022	0.198	**0.001**
2020 vs. 2022	0.15	**0.001**			
2021 vs. 2022	0.24	**0.001**			
July vs. September	0.354	**0.001**			

*Note:* Statistically significant differences marked in bold.

**FIGURE 4:**
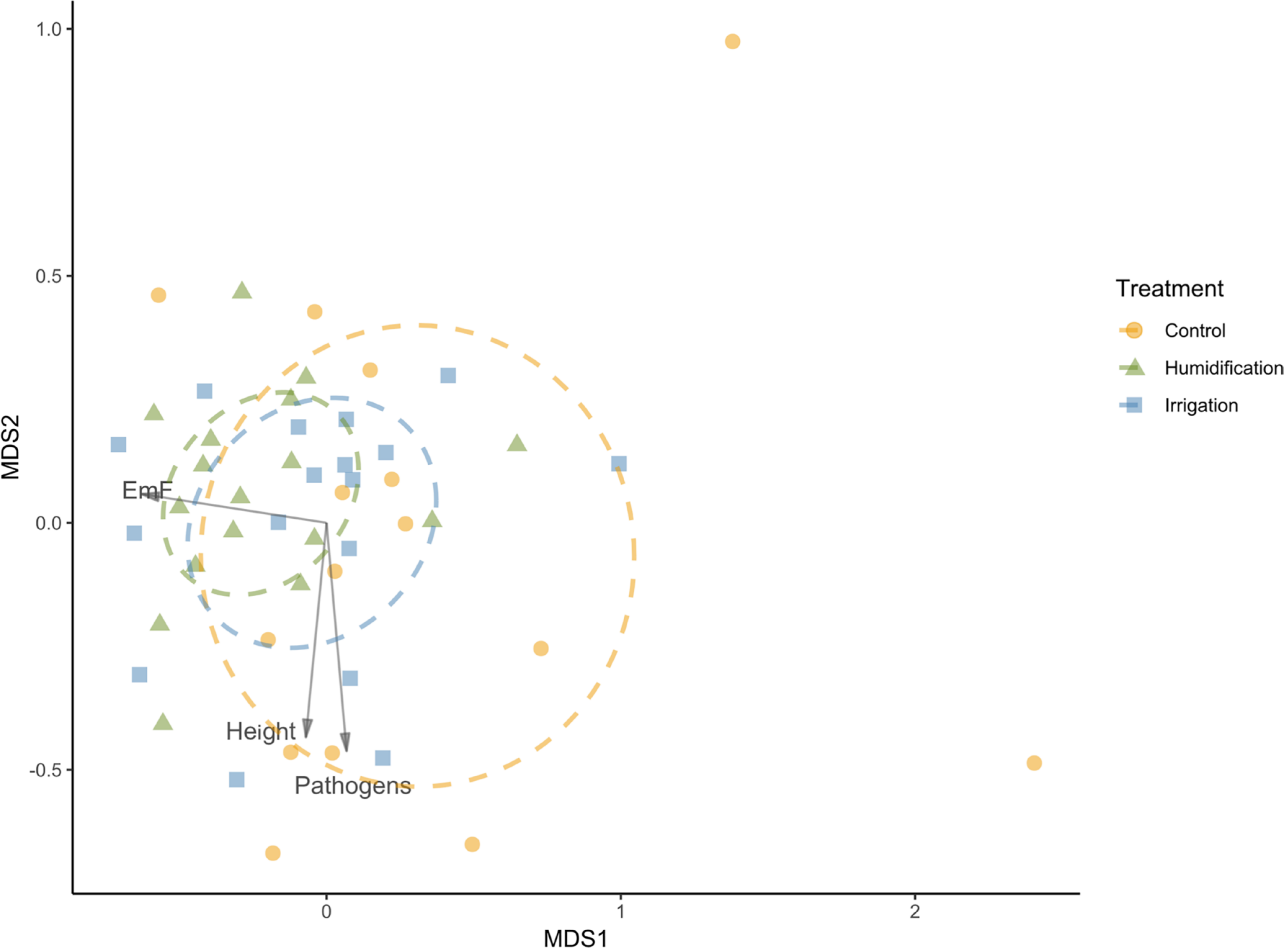
NMDS ordination biplot of fungal communities in silver birch roots, samples taken in October 2021 and 2022, stress = 0.1861. Goodness of fit of various factors to the ordination: Experimental treatment: *r* ^2^ = 0.117, *p* = 0.024; sampling year: *r* ^2^ = 0.327, adjusted *p* = 0.004; EmF—proportion of ectomycorrhizal fungi: *r* ^2^ = 0.513, adjusted *p* = 0.004; proportion of pathogenic fungi: *r* ^2^ = 0.287, adjusted *p* = 0.004; tree height: *r* ^2^ = 0.226, adjusted *p* = 0.007. The dashed line ellipses represent the 95% confidence area of fungal communities in the treatments.

In root fungal communities, there was a smaller difference between the treatments (ANOSIM *R* = 0.069, *p* = 0.001); pairwise comparisons revealed a difference between communities in H and C plots, but no differences between H and I or between I and C (Figure 4). There was also a significant difference in root fungal communities between 2021 and 2022, with ANOSIM *R* = 0.198 and *p* = 0.001 (Table 1). In the NMDS ordination biplot, taxonomic composition variation in root fungal communities was strongly correlated to the proportion of ectomycorrhizal species in samples, whereas it was not correlated with fungal species richness (Figure 4). The proportions of pathogenic fungal taxa also correlated positively with tree height.

### 3.3 Fungal OTU Richness and Functional Groups

**FIGURE 5:**
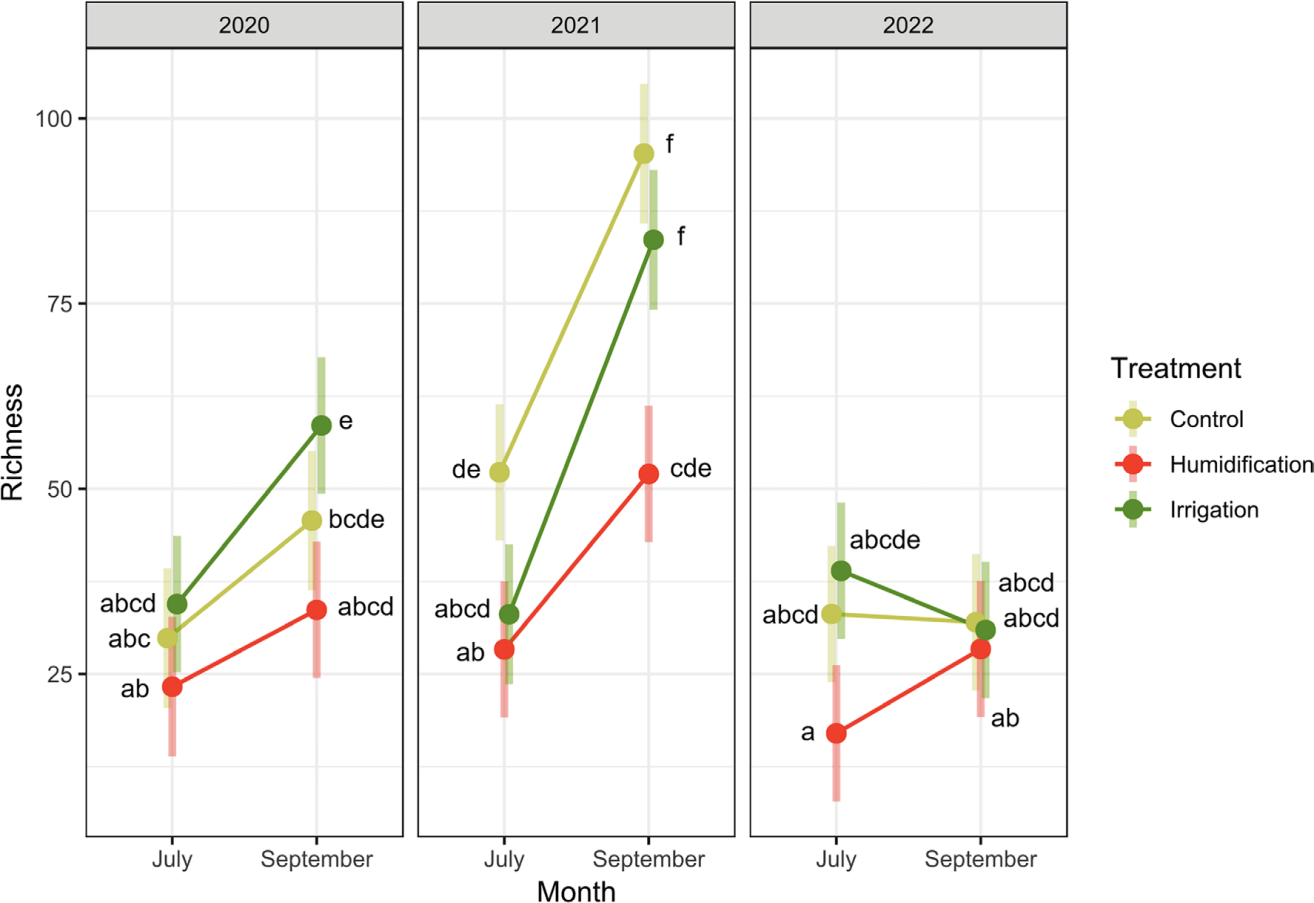
Variation in fungal species richness in birch leaves depending on experimental treatment, sampling month and year as fixed factors (group means with 95% CIs). The lowercase letters near the means denote significant differences (*p* < 0.05) between the groups based on the Tukey test (groups that do not share any letters are significantly different from each other).

**FIGURE 6:**
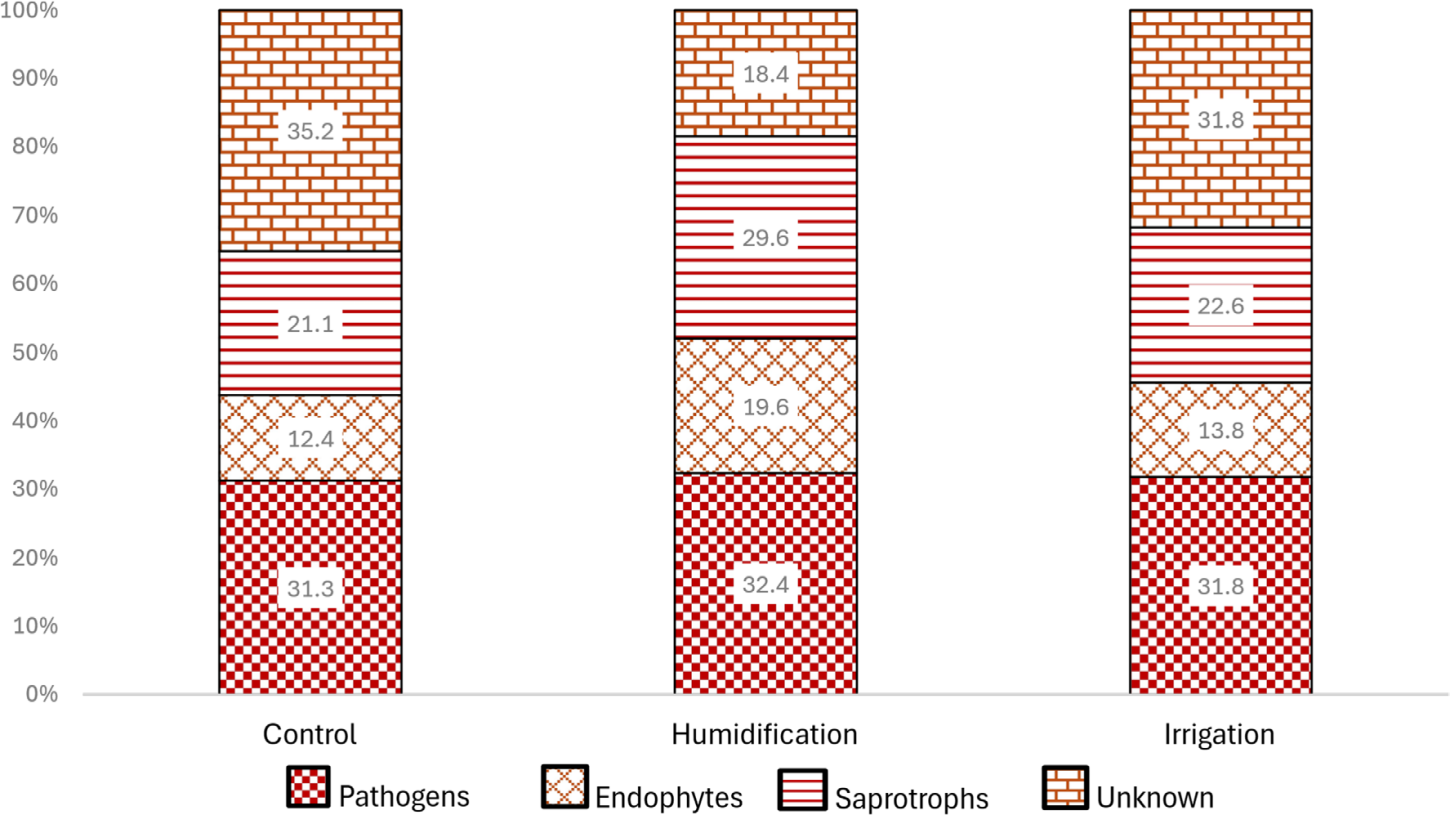
Proportions of fungal functional groups in birch leaves in different experimental treatments over three sampling years.

**FIGURE 7:**
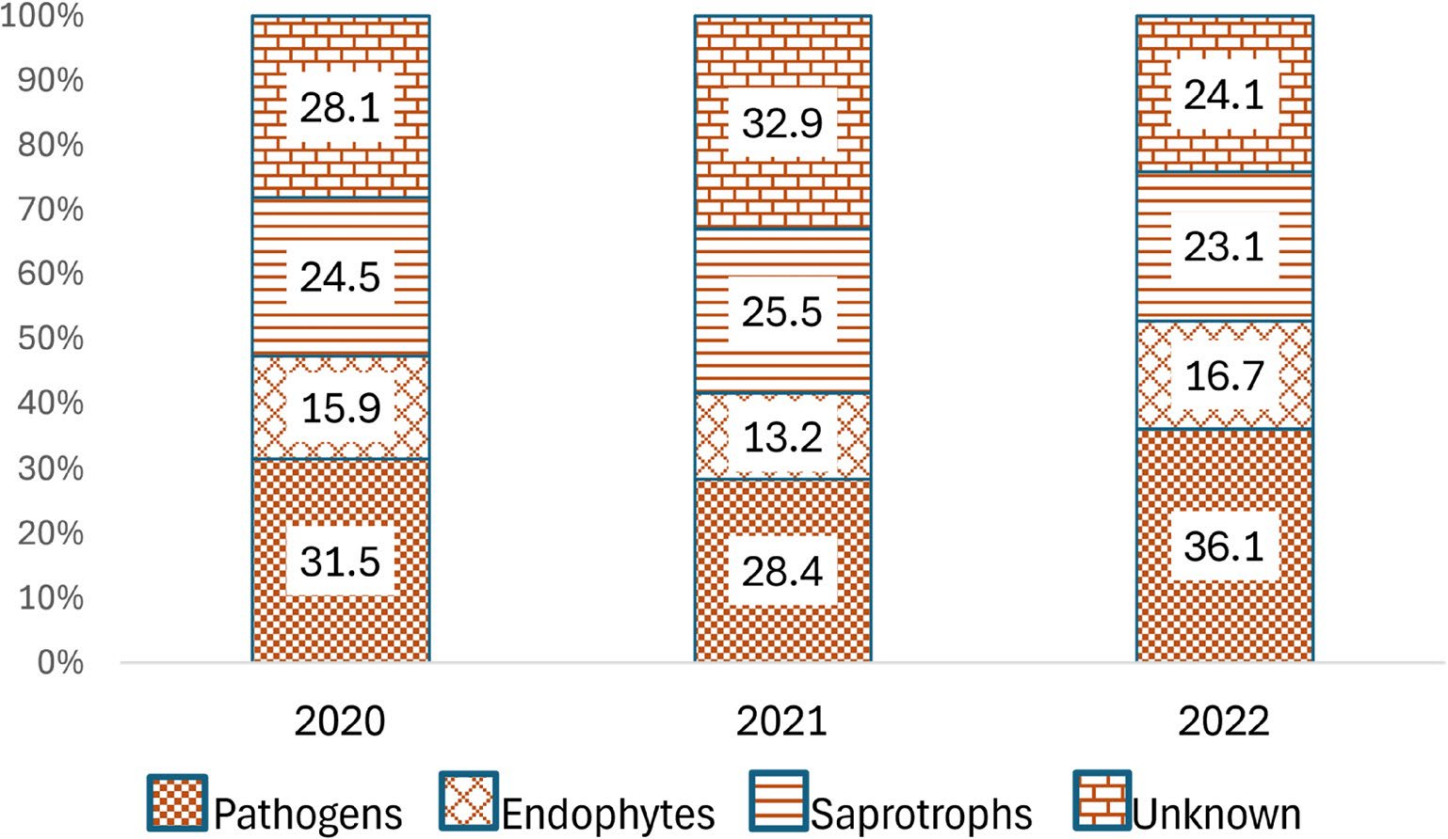
Proportions of fungal functional groups in birch leaves in different years.

Leaf fungal OTU richness was influenced by the combination of all the tested fixed factors—the sampling year, month, and the experimental treatment (Table 2, Figure 5). The proportions of saprotrophic, endophytic fungi and fungi with unknown ecological functions were influenced by the experimental treatment and by the time of sampling. The proportion of pathogenic fungi depended only on the time of sampling (Table 2). Saprotrophic fungi and fungi with endophytic capabilities showed a preference for the H treatment (Figure 6), whilst the proportion of sequences not assigned to any functional group (OTUs not identified to genus or species level) was lower in the H treatment; all the whilst, it was highest in 2021 and lowest in 2022 (Figure 7).

**TABLE 2:** Summary of mixed‐effects ANOVA testing the various fixed effects on fungal OTU richness and functional group proportions in birch leaves.

Fixed terms *p*	DF_1_	Richness	DF_2_	Pathogen %	Saprotrophic %	Endophyte %	Unknown%
Treatment	2/6	**0.020**	2	0.959	**< 0.001**	**< 0.001**	**< 0.001**
Year	1/294	**< 0.001**	2	**< 0.001**	**0.003**	**< 0.001**	**< 0.001**
Month	2/294	**< 0.001**	1	**0.008**	0.297	0.786	**0.004**
Treatment*Year	2/294	**< 0.001**	4	0.587	0.834	0.753	0.201
Treatment*Month	4/294	0.283	2	0.766	0.530	0.914	0.879
Year*Month	2/294	**< 0.001**	2	**0.014**	0.118	**0.025**	**< 0.001**
Treatment*Year*Month	4/294	**0.002**	4	0.128	0.528	0.592	0.307
Random terms variance							
Plot		6.412		0	0.0	< 0.001	0
Tree		0		0	0	< 0.001	0
Residual		329.4					

*Note:* DF_1_—numerator/denominator (rounded to the nearest whole number) degrees of freedom of OTU richness. DF_2_—degrees of freedom of other response variables. Statistically significant effects are marked in bold. Proportions of fungal functional groups in leaf samples are depicted in Figure 6.

**FIGURE 8:**
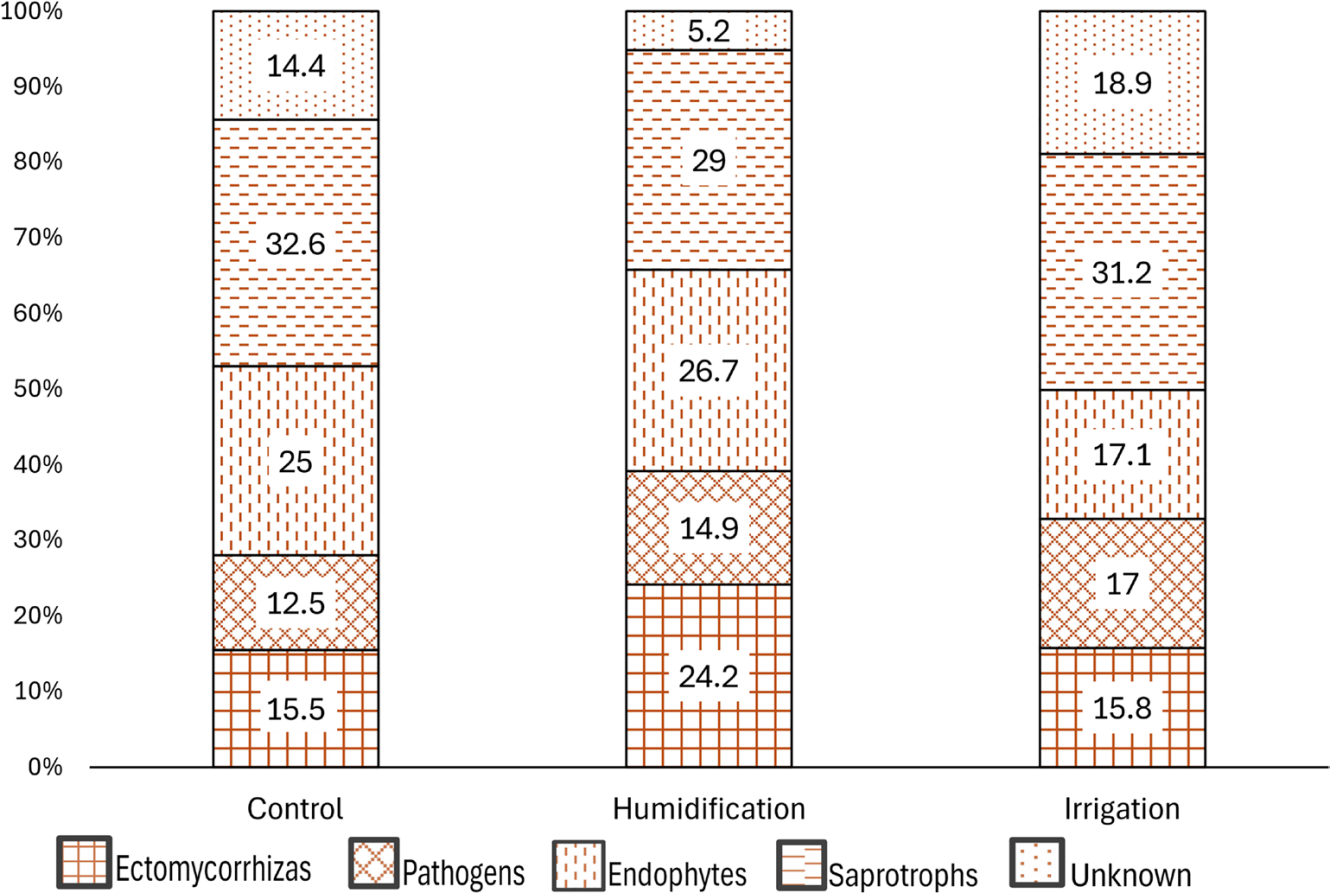
Proportions of functional groups in birch root samples in the experimental treatments over two sampling years.

**FIGURE 9:**
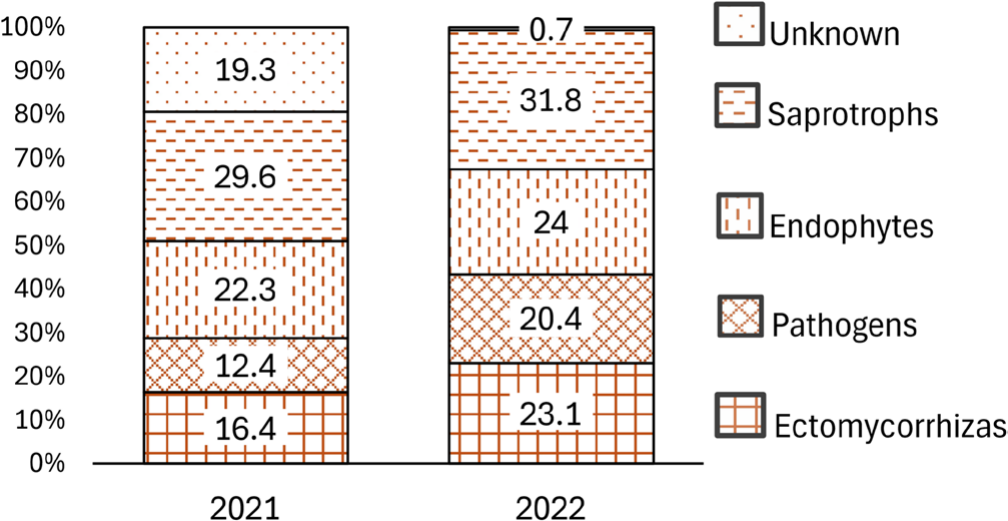
Proportions of functional groups in birch root samples in different sampling years.

In birch root samples, experimental treatment was not a significant factor influencing fungal species richness (*p* = 0.315); sampling year significantly influenced species richness (*p* = 0.003), being higher in 2021; there was also an interaction effect between the two factors (*p* = 0.033) (Table 3). The experimental treatment did not significantly influence the proportions of any functional fungal guilds in the roots, even though there was a decrease in the proportion of ectomycorrhizal fungi under irrigation (Figure 8). Mycorrhizal and pathogenic fungi became more abundant from 2021 to 2022, whilst the number of sequences not identified to at least genus level (in the Other/Unknown category) decreased (Figure 9).

**TABLE 3:** Summary of mixed‐effects ANOVA testing the various fixed effects on fungal species richness and functional group proportions in birch roots.

Fixed terms	DF_1_	Richness	DF_2_	Pathogen %	Saprotrophic %	Endophyte %	Mycorrhiza %	Unknown%
Treatment	2/5	0.315	2	0.067	0.699	**0.004**	0.157	0.318
Year	1/39	**0.003**	1	**0.001**	0.488	0.965	**< 0.001**	**0.001**
Year*Treatment	2/39	**0.033**	2	0.137	0.490	0.907	0.488	**0.006**
Random terms variance								
Plot		24.96	0.009	0	0	0	0	
Residual		342						

*Note:* DF_1_—numerator/denominator (rounded to the nearest whole number) degrees of freedom of OTU richness. DF_2_—degrees of freedom of other response variables. Statistically significant effects marked in bold. The differences in proportions of functional groups by treatment and year are illustrated in Figures 8 and 9.

### 3.4 Indicator Species Analysis

In birch leaf samples, 143 taxa (out of the total of 755) were indicator taxa of one or two treatments. The list of indicator species by experimental treatment is provided in Table S1. Twenty‐six taxa were indicators of C treatment, 15 of H treatment, and 29 of I treatment. Sixty‐eight taxa were indicative of C and I treatments combined; 5 taxa were indicators of H and I. Most of the indicator taxa were relatively uncommon, with 62% of all indicator OTUs being present in less than 15% of samples from their preferred treatment. There were 39 taxa associated with sampling year 2020, 122 taxa associated with 2021, and 5 with 2022.

The relative abundances of indicator taxa were also remarkably different between the experimental treatments. For example, amongst indicator taxa of C + I treatments, there were 10 species present in more than 50% of all samples collected from these treatments. All but one of these taxa had at least 95% of their occurrences happen in these two treatments, being nearly absent in H. By contrast, the most common indicator species of C treatment, *Taphrina communis*, was present only in 38% of all samples collected from C (82% of its occurrences being in year 2021); the most common indicator species in I was present only in 10% of samples collected from I. *Symmetrospora coprosmae*, the most common indicator species in H, was present in 62% of all samples collected from H plots. Several *Taphrina* species showed a strong preference to grow under normal atmospheric conditions and to avoid elevated air humidity. However, this did not include *T. betulina*.

In root samples, there were few indicator taxa: 2 in C treatment (unidentified fungus and *Paxillus involutus*), 4 in I (*Oliveonia pauxilla*, an unidentified ascomycete, a *Gorgomyces* sp. and *Vishniacozyma victoriae*) and one in H + I (*Tuber* sp.); 7 in total. There were 24 taxa significantly associated with the year 2021; four were associated with 2022.

## 4 Discussion

In this study, we investigated foliar and root fungal communities of silver birch using high‐throughput sequencing technique and established that air humidity is an important factor influencing foliar fungal community composition. Contrary to our hypothesis, we did not find any evidence of elevated air humidity making foliar fungal communities any more species‐rich nor more detrimental to leaf health and vitality.

The most common fungi in leaf samples were a Dothideomycetes OTU, possibly an *Endosporium* spread from the aspens (Tsuneda et al. 2008) growing in the buffer zone, that was present in 91% of samples, *Cladosporium herbarum* , a ubiquitously spread fungus growing on many different host species and surfaces (Schubert et al. 2007), present in 89% of sampled leaves, and *Venturia ditricha*, present in 87% of samples, a very widespread fungus on birches everywhere (Helander et al. 2007), with slight pathogenicity. We did not detect any presence of *Cryptosporella betulae*, which has been earlier reported to cause damage in elevated air humidity treatments in our study site (Hanso and Drenkhan 2010; Sellin et al. 2015).

### 4.1 Differences in Communities Between Treatments, Seasons and Years

The alteration in fungal communities of birch leaves caused by the experimental treatment persisted throughout the three years of sampling. Altogether, it accounted for 6.5% of community composition variation. The fungal communities in C and I treatments were alike each other whilst the ones in H were different from them. This suggests that increased air humidity, not increased soil moisture, would be the driver behind possible changes in foliar fungal communities caused by predicted rise in precipitation (Anger‐Kraavi et al. 2021). The effect of treatment on community composition was weaker than those of year and month of sampling, which accounted for 9.8 and 10.8% of the total variation, respectively. The large differences between the sampling months can be explained by the fact that the foliage is constantly exposed to spores floating in the air and over time more successful inoculations happen, as well as the fact that with ageing, the resistance of the leaf to new fungal colonisations declines (Häffner et al. 2015). This gives opportunity to many more fungal species to colonise the leaf. Thus, the species richness generally increases with foliage age (Sieber 2007), although in our case the increase was not always statistically significant due to large variances in species richness and co‐effects of various factors. In our study, there was also an exception to this in 2022, as that year we did not detect significant rise in taxonomic richness from July to September.

In this study, the yearly variation between fungal communities seemed to be more random than seasonal variation, most likely being driven by differences in weather (Crandall and Gilbert 2017; Pakpour et al. 2015), both during the winter and the growing period, success of overwintering and sporulation of endophytic fungi. Fungal spore release is often driven by high air humidity and precipitation (Crandall and Gilbert 2017; Huffman et al. 2013), and most fungi seem to produce spores during the wetter and moderately cool seasons. Higher leaf wetness is also believed to enhance the germination of fungal spores on leaves (Bradley et al. 2003; Crandall and Gilbert 2017). However, fungal spores persist in the air for a longer time in dry conditions, as precipitation may force a lot of spores down onto the ground (Pakpour et al. 2015), although the overall fungal spore abundance in the air peaks in the wet season (Crandall and Gilbert 2017). The misting applied in the humidification treatment may also have taken a noticeable part of spores out of the air, working similarly to rainfall, although elevated air humidity by itself should contribute to high spore concentrations. This may in part explain the lower fungal species richness that was sometimes observed in the elevated air humidity treatment. Still, the effect of elevated air humidity on foliar fungal richness remained inconsistent throughout the study period, as there may have been many other undetectable processes, as well as a degree of stochasticity influencing fungal community assembly. It is still impossible for us to make any definitive predictions on whether climate change will have any impact on the taxonomic diversity of foliar fungi.

Elevated air humidity also affects foliar mycobiome in another way. Under reduced vapour pressure deficit caused by increased air humidity, leaf glandular trichome density decreases and the composition of chemical defence compounds changes, which may decrease plant resistance to fungal colonisation (Lihavainen et al. 2017) and make it easier for endophytic fungi to penetrate into the leaf. We did find that fungi with endophytic capabilities, those that are able to grow inside the leaf tissue, made up a larger proportion of all present fungi in H treatment. This also suggests that elevated air humidity may have a larger negative impact on fungi growing on the leaf surface, and that leaf tissues may provide some protection to the fungus from the outside environment.

5.3% of variation of birch root fungal communities was attributable to experimental treatment; the yearly variation in sampled communities was slightly smaller—4.5%. The more stable conditions below ground may somewhat ameliorate the influence of aboveground climate, but nevertheless, the effect of altered atmospheric conditions turned out to be a significant factor in shaping fungal communities in below‐ground plant organs, without influencing taxonomic richness (Table 3). Elevated air humidity slows soil water depletion because plants take up less water from the soil as transpiration decreases (Kupper et al. 2011) and thus soil contains more moisture. During a previous rotation of trees in this experiment it had been shown that elevated air humidity alters ectomycorrhizal fungal communities of birch roots by causing a shift towards dominance of hydrophilic morphotypes (Parts et al. 2013). We detected a shift in the whole root fungal communities due to increased air humidity; however, we detected only a few indicator species of any treatment, suggesting that either a vast majority of root‐inhabiting fungi are rather tolerant to variation in moisture or that the experimental treatment did not change soil water status enough to inhibit many of the present species from being able to thrive there. Air humidification induced a greater shift in the fine root fungal community composition than soil irrigation compared to the control. Whilst air humidification also slightly increases soil moisture through reduced transpiration, fine root fungal communities are exposed to two elevated factors: soil moisture and air humidity. These two factors combined exert a cumulative influence on fine root fungal communities, having caused a greater shift than soil irrigation alone.

### 4.2 Indicator Species

Indicator species analysis of birch leaf fungal communities revealed over 140 different taxa significantly associated with one or two of the experimental treatments (Table S1). There were no taxa that were correlated with C and H treatments simultaneously; there were four taxa associated with H and I treatments that were rare in C. Taxa that were indicative of both C and I treatments can be viewed as taxa not well suited to grow in elevated atmospheric humidity conditions due to environmental filtering of spores or reduced ability to germinate.

Amongst species correlated with two of the experimental treatments, C and I were by far the most common pairing, with 68 taxa being indicative, making up nearly half of all indicator taxa. Thus, these taxa were largely absent in H treatment, signalling that their fitness or competing ability may be hindered by elevated air humidity. At the same time, there were 15 indicator taxa in H plots. All in all, indicator species analysis suggests that most of the foliar fungal species are not negatively influenced by increased air humidity; however, around 16% of all species present in our sampling data were. The taxa preferring to grow in normal atmospheric humidity conditions outnumber those that prefer elevated air humidity.

Of potentially important biocontrol agents, the genus *Vishniacozyma*, which contains endophytic yeasts, displayed a preference for increased humidity, with 3 of the 6 species detected by DNA analysis being indicator species in H treatment and one in H + I. In root samples, *V. victoriae*, the only present member of the genus, was an indicator species of irrigation treatment, being completely absent in other treatments. Some *Vishniacozyma* species, particularly *V. victoriae*, have been recently reported to be biocontrol agents against some plant pathogenic fungi (Nian et al. 2023; Sepúlveda et al. 2022; Vujanovic 2021) such as *Botrytis cinerea*. In our samples, *B. cinerea* was in fact an indicator species of C and I treatments, albeit not the strongest, with 85% of its occurrences being in these two treatments, supporting that interaction between these taxa may be possible. On the contrary, *Fusarium*, a causal agent of necrotrophic blight, which has also been previously reported to be hindered by the presence of *Vishniacozyma* (Vujanovic 2021), was widespread in every treatment.

Several *Taphrina* species, mild pathogens of birch and other trees, were largely absent in leaf samples from H plots. This, however, did not include the causal agent of witches' broom, *T. betulina*, one of the better‐known pathogenic fungi inhabiting different *Betula* species.

We also observed noticeable changes in fungal species composition in birch leaves between sampling months. Fourteen taxa were indicative of July, becoming rare or completely disappearing by September; 108 taxa were indicators of September samples, most common amongst them *Phyllactinia guttata*, which causes birch leaf spot disease on ageing leaves (Adamska 2012), *Hymenula cerealis*, which parasitises some graminoid species (Hawksworth and Waller 1976), *Ramularia* and *Alternaria angustiovoidea*. In fact, *P. guttata* became so frequent in September samples that it produced more than twice as many sequences as any other fungus and it appears to be one of the main fungal drivers of birch leaf ageing and decay.

## 5 Conclusions

We found that elevated air humidity but not increased soil moisture altered foliar fungal communities in *Betula pendula* . Therefore, we expect that increasing precipitation predicted for northern Europe will influence foliar fungal communities through rising air humidity, but not through increasing soil moisture. Our results also suggest that most foliar fungi of silver birch are rather resilient to increasing air humidity. Still, there were numerous taxa, about 16% of all the taxa we detected, that were negatively affected by increasing air humidity. There were considerably fewer taxa that showed a preference for a more humid environment in mesic northern forests that we researched. Most of the taxa significantly influenced by the experimental treatment were rather uncommon, with 62% of those being present in less than 15% of the samples of their preferred treatments. We found no evidence that increasing humidity could decrease the taxonomic richness of foliar fungi in silver birch. In terms of future health of forests, it may be important that we did not detect an increase in abundance of fungal pathogens under increased air humidity or soil moisture conditions; however, there were some shifts in proportions of other fungal guilds caused by these changes.

## Author Contributions

**Mihhail Brodski:** conceptualization, investigation, writing – original draft, writing – review and editing, visualization, formal analysis, data curation. **Arvo Tullus:** conceptualization, investigation, funding acquisition, writing – original draft, writing – review and editing, supervision, project administration, formal analysis. **Ahto Agan:** conceptualization, investigation, writing – original draft, writing – review and editing, methodology, formal analysis, data curation, supervision, project administration. **Kalev Adamson:** conceptualization, writing – review and editing, resources, methodology. **Rein Drenkhan:** conceptualization, investigation, writing – review and editing, resources, methodology, funding acquisition, project administration. **Katrin Rosenvald:** investigation, writing – review and editing, methodology. **Arne Sellin:** conceptualization, funding acquisition, writing – review and editing, project administration, supervision.

## Ethics Statement

The authors have nothing to report.

## Conflicts of Interest

The authors declare no conflicts of interest.

## Supporting information


**Table S1.** Results of indicator species analysis grouped by experimental treatments.

## Data Availability

The data that support the findings of this study are openly available in GenBank at https://www.ncbi.nlm.nih.gov/sra/PRJNA1211857, reference number PRJNA1211857.
